# P-2171. Genomic epidemiology of Human Respiratory Syncytial Virus in vaccinated and unvaccinated adults in Atlanta, Georgia, during the 2024–2025 season

**DOI:** 10.1093/ofid/ofaf695.2334

**Published:** 2026-01-11

**Authors:** Said Rachida, Henok A Tafesse, Mackenzie Duford, Hannah Cayla C Dakanay, Alaa Ahmed, Anne Piantadosi

**Affiliations:** Emory University, Atlanta, Georgia; Emory University School of Medicine, Atlanta, Georgia; Emory University, Atlanta, Georgia; Department of Pathology and Laboratory Medicine, Emory University School of Medicine, Atlanta, Georgia; Emory University School of Medicine, Atlanta, Georgia; Emory University School of Medicine, Atlanta, Georgia

## Abstract

**Background:**

Human respiratory syncytial virus (RSV) is a leading cause of lower respiratory tract infections in high-risk individuals. The recent introduction of vaccines and prophylactic monoclonal antibodies is expected to reduce the clinical and public health impact of RSV but may also create selective pressure for immune escape among circulating viruses.

**Methods:**

We evaluated clinical and demographic data from 183 adult patients who presented to Emory University Hospital between September 21, 2024, and March 26, 2025. We collected residual upper respiratory swab samples and performed subgroup-specific RT-PCR followed by viral whole-genome sequencing using the xGen™ Respiratory Virus Amplicon Panel and phylogenetic analyses using Nextstrain.

**Results:**

Among 183 adult patients, the median age was 61 (range: 18–99), and 69.5% were female. Most patients (97.8%) were symptomatic; 12.9% were hospitalized, 2.2% required ICU care, and 2.8% died. Of 83 vaccine-eligible patients, only 17 (20.5%) had been vaccinated. RT-PCR identified 84 RSV-A and 91 RSV-B infections. Fever was more common in RSV-B (76% vs. 61%, p = 0.05); other outcomes were similar. Viral whole-genome sequencing achieved >95% coverage in 108 samples (60%). RSV-A sequences exhibited high clade and lineage diversity. The majority (35%) belonged to lineage A.D.3.1, a descendant of clade A.D.3, while other sequences were distributed across clades and lineages including A.D.1, A.D.1.5, A.D.1.6, A.D.3, and A.D.5.2, each representing 5–25% of cases. RSV-B sequences were predominantly from lineage B.D.E.1 (55%), a major lineage within clade B.D.E, with additional lineages including B.D.4.1, B.D.4.1.1, B.D.E.1.4, and B.D.E.5 detected in 5–20% of samples. Ongoing analyses will examine clinical and viral genomic differences between vaccinated and unvaccinated individuals.

**Conclusion:**

This study revealed substantial genetic diversity in circulating RSV strains and highlighted low vaccine uptake among eligible individuals. These findings underscore the ongoing need for viral genomic surveillance to monitor emerging variants and guide public health strategies as preventive measures become more common.

**Disclosures:**

Anne Piantadosi, MD, PhD, Delve Bio: Advisor/Consultant|Delve Bio: Board Member

